# Immediate direct-to-implant breast reconstruction: A single center comparison between different procedures

**DOI:** 10.3389/fsurg.2022.935410

**Published:** 2022-07-18

**Authors:** Francesco Klinger, Andrea Lisa, Alberto Testori, Stefano Vaccari, Valeria Bandi, Valerio Lorenzano, Marco Klinger, Corrado Tinterri, Valeriano Vinci

**Affiliations:** ^1^ Department of Medical Biotechnology and Translational Medicine BIOMETRA, Plastic Surgery Unit, BIOMETRA, Humanitas Clinical and Research Hospital, Reconstructive and Aesthetic Plastic Surgery School, University of Milan, Milan, Italy; ^2^ Thoracic Surgery Department, Humanitas Research Hospital and Cancer Center, Milan, Italy; ^3^ Breast Surgery Department, Humanitas Research Hospital, Milan, Italy; ^4^ Department of Biomedical Sciences, Humanitas University, Milan, Italy; ^5^ Humanitas Clinical and Research Center-IRCCS, Milan, Italy

**Keywords:** breast reconstruction, breast implants, subcutaneous breast reconstruction, direct to implant (DTI), prepectoral breast reconstruction, acellular dermal matrix

## Abstract

**Background:**

The increased incidence of conservative mastectomy operations (nipple- and skin- sparing) has increased the frequency of immediate breast reconstructions (IBR). In order to guarantee patients the best possible aesthetic outcome, the least chance of complications and moreover, the least postoperative pain, the technique with prepectoral prosthetic pocket was recently reconsidered with the use of ADM. This is the first study using Fortiva^®^ in prepectoral breast reconstruction, and it compares the outcomes of three different patient populations (undergoing retromuscular, prepectoral and prepectoral reconstruction with ADM). The authors suggest that prepectoral breast reconstruction with ADM may bring benefits compared to the current standard technique (retromuscular) as well as compared to the prepectoral reconstruction without ADM.

**Methods:**

Retrospective data analysis of patients who underwent mastectomy followed by immediate breast reconstruction with silicone implants (DTI), performed by a team of breast surgeons and plastic surgeons. Logistic factor regressions were performed in order to investigate the effects of the three different intervention techniques on the incidence of complications. Fisher's exact test was used to analyze the differences in the occurrence of each complication. Mann Whitney test was used to compare the averages of referred pain. A *p* value <0.05 was considered significant.

**Results:**

A total of 67 patients underwent DTI reconstruction, of which 43 with retromuscular prosthesis, 13 prepectoral and 11 prepectoral with ADM. We found a significantly lower incidence of surgical complications with ADM, exclusively in comparison with retromuscular reconstruction (*p* = 0.028). It emerges prepectoral reconstruction with ADM involves significantly less visibility of the implant than both the prepectoral surgery without ADM (*p* = 0.013) and the retromuscular technique (*p* = 0.029). Finally, postoperative pain referred at twelfth month is significantly less relevant in the group with prepectoral prosthesis and ADM, both in the group with retromuscular (*p* < 0.001) and prepectoral without ADM (*p* = 0.001).

**Conclusions:**

This study demonstrates that immediate prepectoral breast reconstruction with ADM is a safe and reliable technique, able to exceed some type of limits imposed by prepectoral reconstruction. Moreover, it provides benefits if compared to the current standard technique. In the future, this technique could also be added to it, after a proper selection of patients in pre- and intraoperative time.

## Introduction

Implant-based breast reconstruction (IBR) is the most common procedure to reconstruct patients affected by breast cancer (1). Whether performed as a staged or a single operation, the prosthesis may be placed in the subpectoral plane, secured along the inferolateral pole by means of a biomaterial adjunct such as Acellular Dermal Matrix (ADM) or a mesh (biological or synthetic) in order to reinforce the front wall of the pocket, thus obtaining greater implant safety and better aesthetic results (2–4).

Currently, the standard one-time IBR technique consists in the placement of the prosthesis in the submuscular pocket since prepectoral reconstruction was discarded at the time where it was often followed by serious complications. However subsequent improvements to prosthetic implants proved that these consequences were attributable to the rudimentary technology of prostheses, and not to the surgical technique itself (5–7). The prepectoral placement of the implant has been re- evaluated only recently, proving to be more valid in terms of aesthetic outcomes and personal satisfaction of patients, while the incidence of complications, compared to retromuscular reconstruction, is almost overlapping (8–12). Even our center followed this trend, first using the retropectoral approach, then prepectoral and more recently, when it turned out to be a safe and efficient technique, the prepectoral approach with ADM (13).

In this regard, we decided to carry out a comparative retrospective study in order to analyze our early experience with prepectoral breast reconstruction with and without ADM compared with traditional retromuscular direct to implant breast reconstruction, recording the possible onset of complications and evaluating the post-operative course in these three groups of patients.

We conducted an analysis of multiple parameters associated with immediate single-stage retromuscular, prepectoral and prepectoral breast reconstruction using ADM in a cohort of patients from a single center.

## Patients and methods

### Data collection

Institutional review board approval was obtained for retrospective chart review of consecutive, direct-to-implant breast reconstruction with silicone implants cases performed at the Humanitas Research Hospital in Milan from October of 2018 to September of 2020. We used textured Mentor CPG (teardrop-shaped), Mentor MP (round) and Allergan implants, size range 125–415 cc. Patients included in the study underwent mastectomy and direct-to-implant reconstruction with our plastic surgery team. All surgeons included in the study are currently active at the institution. The number of cases contributed by each surgeon in the prepectoral and retromuscular groups were matched to minimize bias from surgeon technique or preferences. The following clinical characteristics were recorded: age, BMI, history of smoking, type of chemotherapy (neoadjuvant or adjuvant), type of mastectomy (nipple-sparing vs skin sparing), axillary management (sentinel node biopsies and/or axillary dissection), breast implant characteristics type (shaped or round).

The following post-operative complications were identified: infection, seroma, hematoma, wound dehiscence and capsular contracture. In agreement with the WHO, infection was defined as the development of local or systemic manifestations such as fever (body temperature >38°C), local erythema, tumefaction, implant exposure and/or purulent collection, potentially associated with pain. Seroma was defined as a localized accumulation of serous fluid that was clinically evident by palpation, requiring aspiration. A hematoma was defined as any postoperative collection of blood, minor or major whether evacuated or not. Wound dehiscence is a reopening of a surgical incision either internally or externally. Capsular contracture was defined as the physical distortion and elevation of the implant breast reconstruction. It was defined by either of Baker grades (14).

The aesthetic outcome has been blinded assessed using standardized 12-month postoperative photos by three qualified plastic surgeons considering the frequency (presence/absence) of the following conditions: implant visibility, wrinkling, implant dislocation and scar retraction. Patients were aware of the type of reconstruction performed. Visibility and palpability of the implant can occur because the envelope is thin, an excessive volume, non cohesive content, or for cutaneous aging. Wrinkling is defined as skin folding often secondary to collapse of the upper pole. Implants dislocation is a shifting of the implants over time in an incorrect position. Scar retraction is the result of a contractile wound-healing process occurring in a scar that has already been re- epithelialized and adequately healed.

Pain at the surgical site at twelve months from surgery was inspected by the Visual Analogue Scale (VAS) (15).

**Figure 1:**
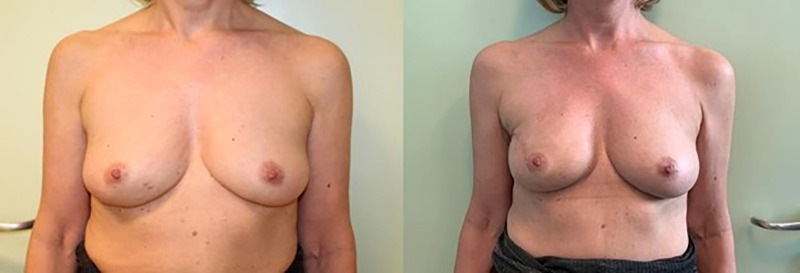
IBR with retromuscular implantation, preceded by monolateral nipple-sparing mastectomy. Pre- (left) and post-operative photos (right).

**Figure 2:**
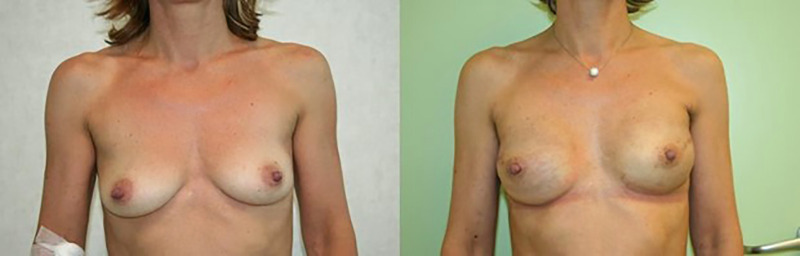
IBR with prepectoral implantation, preceded bilateral nipple-sparing mastectomy. Pre- (left) and post- operative photos (right).

**Figure 3:**
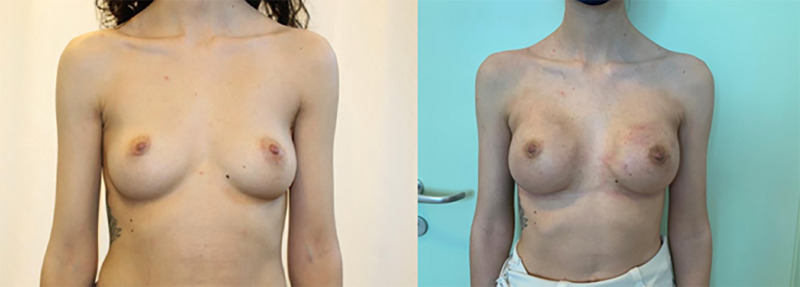
IBR with prepectoral implantation and ADM Fortiva®, preceded by bilateral nipple-sparing mastectomy. Pre- (left) and post-operative photos (right).

The study examines three patient populations: the first, considered as the reference population, consists of patients undergoing immediate reconstruction with retromuscular prosthesis (mono- or bilateral), a technique considered to be the current standard for breast reconstruction (Figure 1); the second one is formed by patients on which prepectoral (mono- or bilateral) breast reconstruction has been carried out without the use of ADM (Figure 2); finally, the third and last population is a group of patients undergoing prepectoral breast reconstruction with the ADM (Fortiva®, RTI Surgical) (16) (Figure 3).

Fortiva® is an acellular dermal matrix of porcine origin, perforated (to allow an easier passage of fluids and a better integration with the tissue), resistant, ready to use (does not need washing in physiological solution), with a constant thickness of 1 mm (which allows comparable and predictable results) and more cost effective than other ADMs (17–19).

A clinical evaluation of the mastectomy flaps was performed to indicate DTI vs expander; within the DTI population, we didn't randomize the patients before assigning them but we adopted consecutive cases to the 3 study groups.

In view of the above, in this study were adopted as exclusion criteria the factors attributable to damage to the microcirculation, or capable of affecting the formation of granulation tissue and healing processes. Therefore, the patients considered unfit for single-stage reconstruction were women with diabetes, in therapy with cortisone drugs, heavy smokers (>10 cigarettes/day), candidates for adjuvant RT and/or axillary dissection (20–23).

### Surgical technique

#### Nipple and skin-sparing mastectomy

Mastectomies were performed through an elliptical incision around the areolar margin. Flaps were raised in all directions superiorly toward the clavicle, medially toward the sternum, inferiorly toward the inframammary fold, and laterally toward the latissimus dorsi. Breast tissue was then dissected from the pectoralis major muscle. In case of nipple-sparing mastectomy underneath the nipple-areola complex, ductal tissue was excised, preserving the nipple itself. The remaining breast tissue was dissected from the pectoralis major muscle. Once the wound was irrigated and hemostasis was confirmed, the plastic surgeon proceeded with the reconstruction portion of the procedure (24, 25).

#### Subpectoral direct-to-implant reconstruction

On completion of mastectomy, subpectoral direct-to-implant reconstruction was performed. The pectoralis major muscle was elevated along the inferior and lateral margin. The implant was placed in the subpectoral complete pocket and then checked for size, shape, and symmetry.

#### Prepectoral direct-to-implant reconstruction with ADM

**Figure 4:**
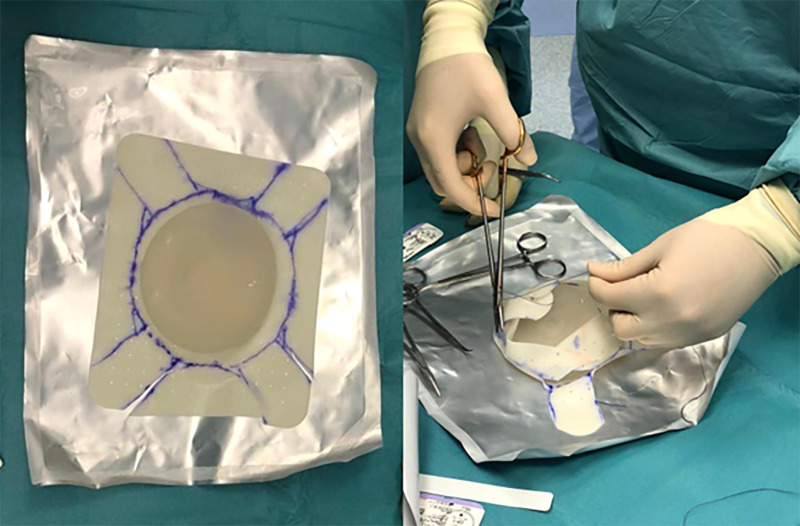
ADM cut to better adapt to the prosthesis and its fixation with stitches.

**Figure 5:**
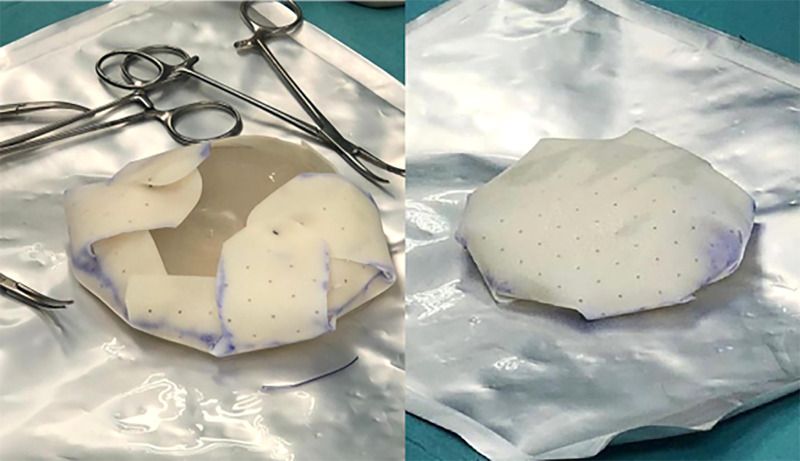
Prosthesis wrapped in ADM ready for implantation.

In case of prepectoral reconstruction using ADM, the prosthesis was previously prepared by wrapping it with the dermal matrix, properly cut and fixed with Vicryl 3-0 suture. The implant was placed in the prepectoral pocket, then checked for size, shape, and symmetry in the seated and lying positions (Figures 4, 5).

### Statistical analysis

The analysis of the occurrence of complications has been divided between surgical and aesthetic complications. Logistical factor regressions were carried out in order to investigate the effects of the three different intervention techniques on the incidence of these complications. The techniques are compared in pairs.

Fisher’s exact test was used to analyze differences in occurrence of each complication as a result of different techniques (infection, dehiscence, capsular contracture, seroma, hematoma and rupture of the implant are the surgical complications; implant visibility, wrinkling, dislocation and scar retraction as aesthetic complications).

Intensity of pain in the mammary area at 12 months from surgery was described in terms of mean, standard deviation and minimum and maximum values. Non-normally distributed variables were assessed through the Mann-Whitney test. Statistical significance was defined as a value of a *p value* <0.05.

## Results

In the 67 consecutive patients undergoing immediate direct-to-implant (DTI) reconstruction analyzed we observed that prostheses were implanted in the subpectoral plane in 43 patients (8 bilateral cases), prepectoral in 13 patients (3 bilateral cases), the remaining 11 patients underwent prepectoral breast reconstruction using ADM (3 bilateral cases). We observe difference in age and number of active and former smokers between the three populations (*p* = 0.001; *p* = 0.024; *p* = 0.024). No difference in BMI (Table 1).

**Table 1:** Clinical characteristics.

Variable	Subpectoral	Non ADM	ADM	*p*
No, patients	43	13	11	
No, breasts	51	16	14	
Age	48.1 ± 7.9	58.2 ± 10.6	46.2 ± 10.3	0.001
BMI	20.4 ± 2.4	21.6 ± 3.0	22.1 ± 2.9	0.097
Active smokers	0	1 (7.7%)	2 (18.2%)	0.024
Former smokers	0	1 (7.7%)	2 (18.2%)	0.024
Chemotherapy ad	13 (30.2%)	1 (7.7%)	3 (27.3%)	0.302
Chemotherapy neo	8 (18.6%)	2 (15.4%)	0	0.365
Nipple-sparing	44 (86.3%)	13 (81.3%)	14 (100%)	0.336
Sentinel lymph node biopsy	36 (70.6%)	11 (68.8%)	13 (92.9%)	0.222

The incidence of complications in the three groups was also assessed (Table 2). Comparing complications between the three groups, though more frequent in subpectoral reconstruction than prepectoral reconstruction with and without ADM (except for seroma, capsular contracture and skin ischemia, which are more frequent in people with prepectoral without ADM, and wound dehiscence, which is slightly more frequent in people with ADM), Fisher's exact test revealed no significant differences between the different groups about individual complications. Therefore, in terms of occurrence of individual complications, the different techniques do not differ significantly (Table 2).

**Table 2:** Surgical complications.

Surgical complications	Subpectoral	Non ADM	ADM	
Skin ischemia	2 (3.9%)	1 (6.3%)	1 (7.1%)	*p* = 0.624
Skin necrosis	1 (2%)	0	0	*p *= 1
Infection	5 (10%)	1 (6.3%)	0	*p* = .0623
Dehiscence of the wound	1 (2%)	0	1 (7%)	*p* = 0.355
Capsular contracture	3 (5.9%)	1 (6.3%)	0	*p* = 1
Seroma	9 (17.6%)	4 (25%)	0	*p *= 0.153
Hematoma	4 (7.8%)	1 (6.3%)	0	*p* = 0.818
Rupture of the implant	2 (3.9%)	0	0	*p* = 1
Prosthetic exposure	0	0	0	*p* = 1
**TOT**	**27**	**8**	**2**	

It is noted that the probability of incurring surgical complications significantly decreases in case of use of the prepectoral with ADM compared to the retromuscular technique (*p* = 0.028).

On the contrary, we observe no significant difference between the retromuscular placement and the prepectoral technique without the use of ADM (*p* = 0.144) or between the prepectoral without ADM and with ADM (*p* = 0.176).

The aesthetic outcome has been evaluated considering the frequency of the following events: implant visibility, wrinkling, implant dislocation and scar retraction (Table 3).

**Table 3:** Aesthetic complications.

Aesthetic complications	Subpectoral	Non ADM	ADM	
Implant visibility	13 (25.5%)	6 (37.5%)	0	*p* = 0.035
Wrinkling	9 (17.6%)	5 (31.3%)	1 (7.1%)	*p* = 0.246
Implant dislocation	7 (13.7%)	0	0	*p* = 0.138
Scar retraction	3 (5.9%)	1 (6.3%)	0	*p* = 1
**TOT**	**29**	**12**	**1**	

By examining the individual aesthetic complications, we have found a significant difference only in the visibility of the implant between the different techniques. Comparing the different techniques, therefore, we observed that the retromuscular techniques and the prepectoral without ADM (*p* = 0.266) do not present significant differences while the prepectoral intervention with ADM results in a significantly lower visibility of the implant both compared to the prepectoral intervention without ADM (*p* = 0.013) and to the retromuscular technique (*p* = 0.029).

Intensity of the pain was assessed through the Visual Analogue Scale (VAS).

In patients with subpectoral reconstruction, the pain reported ranged from 0 to 7 (minimum and maximum), in the prepectoral group from 0 to 2, and in the group with ADM no patient reported pain twelve months after surgery.

From the analysis, it appears that the reported pain is not different as a result of subpectoral or prepectoral without ADM intervention (*p* = 0.441). Instead, significant differences emerge by comparing the retromuscular technique with the prepectoral with ADM (*p* < 0001) and the prepectoral without ADM with the analogous with ADM (*p* = 0.001): in both cases, ADM prepectoral intervention is associated with a significantly lower reported pain index than the other two types.

## Discussion

Immediate implant-based reconstruction is the most common approach for the management of patients with breast cancer. There is a continuous evolution in the techniques: in the 1960s, breast reconstruction with subcutaneous placement of the prosthesis was riddled with postoperative complications, in particular for the high rate of capsular contracture, and largely abandoned by the 1970s (6, 9). Subsequently, we moved on to the retromuscular plane: this option reduces the exposure rate of the prosthesis and subsequent contracture but presents complications such as distant pectoral animation and increased postoperative pain. Recently, prepectoral reconstruction has been revisited in a new light: with the advent of ADM, surgeons could provide additional implant coverage to potentially minimize complications, reduce pain and improve aesthetic outcomes (2, 13, 26–28).

In consideration of the emergence of this technique, new ADMs are being developed while the problem of the costs remains current (29).

Our study was a comparative analysis of subpectoral and prepectoral implant-based reconstruction with and without the use of ADM, adopting Fortiva®, based on data that was retrospectively collected.

Berna et al. were the first to point out the results of prepectoral reconstruction with the use of *Braxon*®, in a series of 25 surgeries underwent by 19 patients. The short-term follow-up demonstrated adequate clinical and aesthetic outcomes (30).

In our study, results similar to Berna's were found, with lower complication rates such as seroma and infection with regard to the prepectoral technique with ADM. In reverse, Chandarana et al. have found comparable outcomes in a series of 154 prepectoral and subpectoral IBRs (31). The implant loss rates were lower in the prepectoral group (4.2%) as compared to the subpectoral group (10.8%), a difference that was not found to be statistically significant. In our study, no prosthetic exposure occurred in any of the 3 study groups.

Casella et al. have highlighted similar outcomes comparing prepectoral and subpectoral single-stage IBR using a synthetic tetanized mesh (TiLOOP®) in a series of 73 mastectomies with an implant loss in one patient and a complication rate of less than 10% in each group (32). Vidya et al. have reported on 100 reconstructions from centres in Europe with an implant loss rate of 2% and satisfactory cosmetic outcomes (33–36).

We conducted an analysis of multiple parameters associated with immediate single-stage retromuscular, prepectoral with and without ADM adoption in a cohort of patients from a single center. This study is the first to use Fortiva® as ADM in prepectoral reconstruction.

In our study, we have noticed a significant decrease of complications in case of prepectoral with ADM compared to the retromuscular technique. ADM increases the coverage of soft tissues of the prosthesis, acts as a scaffold for the formation of new tissue and slows down any fibrotic processes leading to a lower risk of capsular contracture.

Capsular contraction could be one of the leading cause of postoperative pain since in our case series we observed an increased pain in both retromuscular and prepectoral without ADM if compared with prepectoral with ADM adoption (37–39).

We noted that the likelihood of incurring surgical complications in full in the case of using a prepectoral with ADM is significantly lower compared to the retromuscular technique.

However, comparing the single events between the three groups, although more frequent in retromuscular reconstruction than in prepectoral reconstruction, both with and without ADM, such a difference is not statistically significant between the different groups regarding individual complications, confirming the safety of each procedure.

Our analysis showed that the pain reported at 12 months was significantly lower in the prepectoral technique with ADM if compared to the other two techniques so that the formation of periprosthetic tissue could be a leading cause nevertheless another possible explanation could be the better coverage of the prosthesis with a lower fibrosis in the ADM group.

Regarding the aesthetic outcome, prepectoral technique with ADM offers improved cosmetic results with less visible implant if compared with both retromuscular and prepectoral without ADM groups.

Reconstructive outcome is also perceived to be more appealing in prepectoral breast reconstruction since animation deformity is rarely present.

We are convinced that implant visibility and scar retraction can be improved by autologous fat grafting but in our study this was not performed immediately after surgery (37, 38).

Fortiva®, different from some other commercially available ADMs, is not preformed but must be conformed to the implant. We have therefore developed a modeling approach (see picture 4–5) that we believe very effective (33). A problem that can arise during the modeling and bending of the ADM is the formation of local deformations in the edges, which, in our experience, resolves in a month, with the integration of the matrix itself.

We are in an era of managed care and cost-effectiveness. We are convinced that prepectoral reconstruction with ADMs could significantly decrease the probability of incurring surgical complications in case of use of the prepectoral technique with ADMs as compared to the retromuscular technique (22).

Immediate single-stage implant reconstruction using an acellular dermal matrix offers a cost- effective reconstruction with a low complication rate. This may be the procedure of choice in selected patients.

This fact suggests that the cost of the ADM is offset by avoiding a complicated outpatient management of complications and reoperations (40, 41).

Furthermore, from our experience, Fortiva® is cost-effective compared to other ADMs currently on the market.

We acknowledge that our study has several limitations, firstly, the analysis is limited by the small number of patients that met our inclusion criteria. Secondly, our analysis is limited by its retrospective nature and by the fact that patients were not randomized before being assigned to each group but we adopted consecutive cases.

The exclusion criteria are very stringent and in particular include radiotherapy. The main aim of our study was to evaluate the safety of this new ADM; in the future we set ourselves the goal of evaluating the presence of any interaction between prepectoral reconstruction with ADM and radiotherapy.

## Conclusions

This study is the first to use Fortiva® as ADM in prepectoral reconstruction.

To our knowledge, there have been no reports in literature comparing single stage subpectoral and prepectoral with and without the use of ADMs.

By providing a scientific basis to support prepectoral implant placement with ADMs, we could demonstrate a viable option for improving reconstructive outcomes in patients undergoing mastectomy skin and nipple sparing, while reducing costs in terms of hospital stay and surgery.

## Data Availability

The original contributions presented in the study are included in the article/Suplementary Material, further inquiries can be directed to the corresponding author/s.
